# Towards Automated Chicken Monitoring: Dataset and Machine Learning Methods for Visual, Noninvasive Reidentification

**DOI:** 10.3390/ani15010001

**Published:** 2024-12-24

**Authors:** Daria Kern, Tobias Schiele, Ulrich Klauck, Winfred Ingabire

**Affiliations:** 1 Faculty Electronics & Computer Science, Aalen University, 73430 Aalen, Germany; tobias.schiele@hs-aalen.de (T.S.); ulrich.klauck@hs-aalen.de (U.K.); 2 School of Computing, Engineering and Built Environment, Glasgow Caledonian University, Glasgow G4 0BA, UK; winfred.ingabire@gcu.ac.uk; 3 Department of Computer Science, University of the Western Cape, Cape Town 7535, South Africa

**Keywords:** chicken, poultry, livestock, re-ID, individual identification, transformer, dataset, artificial intelligence, machine learning, computer vision

## Abstract

The chicken is the world’s most farmed animal. In this work, we introduce the Chicks4FreeID dataset, the first publicly available dataset focused on the reidentification of individual chickens. We begin by providing a comprehensive overview of the existing animal reidentification datasets. Next, we conduct closed-set reidentification experiments on the introduced dataset, using transformer-based feature extractors in combination with two different classifiers. We evaluate performance across domain transfer, supervised, and one-shot learning scenarios. The results demonstrate that transfer learning is particularly effective with limited data, and training from scratch is not necessarily advantageous even when sufficient data are available. Among the evaluated models, the vision transformer paired with a linear classifier achieves the highest performance, with a mean average precision of 97.0%, a top-1 accuracy of 95.1%, and a top-5 accuracy of 100.0%. Our evaluation suggests that the vision transformer architecture produces higher-quality embedding clusters than the Swin transformer architecture. All data and code are publicly shared under a CC BY 4.0 license.

## Simple Summary

The chicken is the world’s most farmed animal. Artificial-Intelligence-based reidentification offers a stress-free alternative to traditional methods, such as leg bands. In this paper, we introduce the first public dataset designed to reidentify individual chickens in images using artificial intelligence. Additionally, we provide an overview of existing public datasets for reidentifying other animals. On the introduced dataset, we test different artificial intelligence models to visually identify individual chickens. We evaluate two scenarios: using a single image per individual and using multiple images. For scenarios with multiple images per individual, we achieve accuracy rates of between 95.1% and 100%. Additionally, we adapt an artificial intelligence model originally designed for other animals and test its performance on chickens. Our findings reveal that this approach is highly effective when data are limited.

## 1. Introduction

### 1.1. Background

Chickens struggle to recognize other individuals after visible changes are applied to the comb or plumage [1]. Much like chickens are able to use visual cues to differentiate each other, artificial intelligence (AI) is capable of utilizing image or video inputs for reidentification (re-ID) purposes. Animal re-ID, the task of identifying individual animals within one (or sometimes several) species, finds applications in various fields, particularly in livestock management [2,3,4,5,6,7,8,9,10] and in wildlife conservation efforts, where monitoring endangered species is crucial [11,12,13,14]. Re-ID falls into one of two categories: closed set and open set. In closed-set re-ID, all individuals are known from the beginning, and those to be identified can be matched with identities of a predefined set. In open-set re-ID, the identity of the individual in question may not necessarily be part of a predefined set. It is possible to encounter completely new, undocumented individuals. Such individuals must be annotated as a new identity and, upon subsequent encounters, be accurately matched. Visual traits play a pivotal role in animal re-ID within computer vision, serving as essential markers for distinguishing individuals. Conversely, little interindividual variability poses a challenge to the re-ID task. However, the task is complex and extends beyond mere visual cues. Factors such as lighting, perspective, body changes over time, and partially obscured body parts pose additional challenges [15]. The task of re-ID is closely related to tracking, where individuals are detected and tracked across various video frames. During tracking, individuals often need to be reidentified after leaving and re-entering the field of vision. For instance, the challenge of individual chickens disappearing between frames or being occluded in later frames can be effectively addressed by chicken re-ID, which accurately recognizes the returning individual as the same chicken that left the frame.

### 1.2. Motivation

Automated tracking holds great potential in precision livestock farming [16]. These systems may allow for the observation of social structures and behavior, enhance welfare, and lead to more efficient animal management with minimal disruption to the livestock [17]. Chicken welfare assessment is increasingly focusing on individual animals rather than entire groups [18]. To effectively monitor, i.e., behavior, especially when dealing with many animals in a large group, it is crucial to accurately recognize and track each individual [18]. Moreover, EU regulations require individual identification to ensure traceability during disease outbreaks [19]. Traditional methods for re-ID, such as leg bands, wing tags, or backpacks with sensors, can cause significant stress to the animals and have been shown to negatively affect behavior, the immune system, and body weight [20,21]. AI-based re-ID offers a noninvasive and efficient alternative to traditional identification methods. Furthermore, identifying individual chickens is essential for behavioral studies [22], as it plays a crucial role not just in experiment oversight but also in ensuring accurate statistical analysis. The stress induced by traditional methods not only affects the animals’ well-being but also compromises the validity of research findings. AI-based identification maintains the integrity of a study while minimizing the stress on the animals. Moreover, ref. [23] recommends continuing exploring the ethological complexity of chickens in settings that are noninvasive and nonharmful, not only in commercial farming settings but also in more naturalistic settings.

Despite the significant potential of re-ID technologies, there is a notable lack of datasets that form the foundation for such advancements. Notably, as far as we are aware, no publicly available re-ID dataset for chickens currently exists, underscoring the need for development in this area. Public datasets for individual animal re-ID are scarce generally [17,24], in particular well-annotated datasets [8]. The practice of openly sharing data and code should be encouraged to enhance result comparability, but not all research data are currently made public.

### 1.3. Contributions

In broad terms, we performed visual closed-set re-ID of 50 individual chickens. The data used were cut-out crops of the chickens’ bodies, which excluded the background. The dataset was specifically constructed for this task, with images taken from a standing position to emphasize the plumage as the primary identifying feature.

In detail,

i We present a summary of animal re-ID studies and give a comprehensive overview of publicly available datasets.
ii We address the existing gap and introduce the first publicly available dataset for chicken re-ID: Chicks4FreeID. The dataset supports closed -and open-set re-ID, as well as semantic and instance segmentation tasks. We make this thoroughly documented dataset freely accessible to the research community and the public.
iii We evaluate a species-agnostic state-of-the-art model on our dataset through two experiments. In the first experiment, we test the model using its frozen weights, which were not trained on chicken data. In the second experiment, we fine-tune the model to adapt it specifically to our dataset.
iv We train two feature extractors from scratch in a standard supervised manner and test them on our dataset. Both models are based on transformer architectures.
v We perform additional one-shot experiments with all previously mentioned models.
vi Lastly, we make all associated code publicly available to ensure transparency and facilitate further research.

## 2. Related Work

### 2.1. Re-ID

While facial recognition is a prevalent method for reidentifying humans [25], the faces of animals can likewise serve as a means to reidentify individuals, as has previously been demonstrated for rhesus macaque [26], chimpanzee [27], cats [28], lions [29], dogs [30], giant pandas [13], and red pandas [14]. Notably, ref. [31] applied transformer-based similarity learning for chicken re-ID, using the chickens’ heads as the primary identifying feature, with a focus on a uniform group of white laying hens. Additionally, they examined the importance of different head features for visual re-ID, finding that the models prioritized characteristics such as the comb, wattles, and earlobes. However, animals frequently exhibit more distinctive visual traits beyond their faces. For example, natural markings such as stripes [32,33,34,35] and scale patterns [36] have served as prominent identifiers. Also, specific body parts can contribute to distinguishing individuals, such as the fins of dolphins [37] and sharks [38]. Similarly to how fingerprints differentiate humans, the nose prints of dogs have been utilized to uniquely identify individual dogs [39]. However, some studies aimed at identifying animals with little interindividual variability. Species exhibiting minimal or subtle visual distinctions between individuals are, for instance, (polar) bears [40,41] and elephants [42].

### 2.2. State of the Art

To further advance the field and aid the research community, ref. [43] released the WildlifeDatasets toolkit: an open-source toolkit for animal re-ID. It gathers publicly available animal re-ID datasets in one place in an effort to make them more easily accessible and to improve usability. Included are various tools, i.e., for data handling and processing, algorithms relevant to the task of re-ID, pretrained models, as well as evaluation methods. They address the prevailing absence of standardization across the literature and facilitate the comparability and reproducibility of results. Within their work, they also introduce a new state-of-the-art model, the MegaDescriptor, notably the first foundation model for animal re-ID. Likewise, ref. [44] presented an open-source re-ID method initially developed for sea stars, which was successfully extended to seven mammalian species without adjustments. They also reported state-of-the-art results. Moreover, ref. [45] introduced Tri-AI, a system designed for the rapid detection, identification, and tracking of individuals from a wide range of primate species. The system is capable of processing both video footage and still images.

### 2.3. Datasets

A review of the existing resources revealed fewer than 40 publicly available datasets for animal re-ID. This led to the conclusion that a significant number of animal species are not yet covered, including chickens. Birds in general seem to be under-represented in this domain, with only a couple of datasets available [46,47]. In fact, a noticeable focus lies on marine life [48,49,50,51,52,53,54,55,56,57,58]. However, cattle are the most frequently featured species [3,59,60,61,62,63], with much of the data collected by the same group of researchers. Despite chickens being the most widely farmed animal globally, no public re-ID dataset specifically for chickens was found. Table 1 provides a summary of the publicly accessible datasets found for animal re-ID, arranged by year. Each entry details the name of the dataset (“Dataset”), the associated publication (“Publ.”), and species focus (“Species”). “IDs” denotes the number of unique identities present within the dataset. Additionally, the total number of annotated animal instances within all images of each dataset is noted (“Annot.”). An indication (*) of whether the data were derived from video sources is given as well. For ease of access, a direct link to each dataset is provided (“Avail. at”). Although all of the datasets are publicly accessible, some are released under licenses that are relatively restrictive.

## 3. The Chicks4FreeID Dataset

### 3.1. Overview

**Figure 1:**
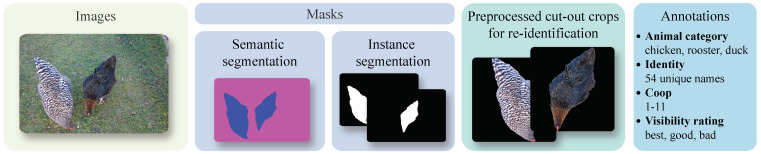
Dataset overview.

The Chicks4FreeID dataset contains top-down view images of individually segmented and annotated chickens. It predominantly features female chickens (hens). For simplicity and to align with colloquial language, we refer to hens as simply “chickens” and male chickens as “roosters”. Some images also feature ducks. Each image is accompanied by a color-coded semantic segmentation mask that classifies pixel values by animal category (chicken, rooster, duck) and background, as well as binary segmentation mask(s) for the animal instance(s) depicted. Additionally, the dataset includes preprocessed cut-out crops (detailed in Section 3.4) of the respective animal instances. The Chicks4FreeID dataset can be utilized for various tasks, including re-ID, semantic segmentation, instance segmentation, and possibly anomaly detection. For each task, a distinct subset configuration was created [64]. Figure 1 gives a first overview of the dataset.

### 3.2. Collection

The data were collected manually, with no images derived from any existing dataset. Eleven private households in southern Germany were visited to photograph chickens. A total of 677 images were captured using two similar models of cameras: the “CyberShot DSC-RX100 VI” and the “CyberShot DSC-RX100 I” from Sony. The resolution of the images stands at 3648×5472 pixels. Each image includes at least one chicken, ensuring no images without chickens are part of the dataset. The animals were allowed to move freely without constraints to prevent distress. As a result, other individuals frequently entered the frame. The chickens were photographed from a standing position, aiming to capture the plumage from a top-down perspective. However, since the animals were free to move, their distance from the camera varied, which affected the view of their plumage. Thus, the resulting images do not show a clean top-town view. Most individuals were photographed outdoors under natural light, except for four individuals, which were indoors in a coop during winter. Data collection took approximately one year. However, all photos of a given individual were taken on the same day.

### 3.3. Annotation

All annotations were manually created by a human annotator using Labelbox [93] under a free educational license.

**Instance Masks** By drawing an almost pixel-perfect polygon outline, a binary instance mask was created for each animal in an image. This process resulted in a total of 1270 instance masks. In each mask, the instance is encoded in white, whereas the black area is considered background. Each instance includes the comb, head, beak, and plumage. In contrast, feet and scattered feathers are classified as part of the background, along with any other visible objects. Feet were intentionally excluded to prevent identification through leg rings. We created instance masks instead of bounding boxes to aid the creation of precise cut-out crops for re-ID. Bounding boxes often include other individuals, which could confuse the model, as well as background elements, which could introduce unintended clues. By using instance masks, we ensure a focus on the animal’s features, avoiding reliance on background information.

**Identities** Each instance was assigned an identity. There were 54 distinct identities. Prior to the photography sessions, the visible features of each animal were meticulously studied and recorded in a notebook. This was conducted to ensure the correct identification of each individual during annotation. Ground truth annotation was therefore performed using expert knowledge, without the use of any algorithms. In cases where the human annotator could not assign an identity, the instance in question was labeled as identity “Unknown”. It is essential to clarify that the label “Unknown” does not imply the presence of a new, undocumented individual. Instead, it represents an unidentified individual from the closed set, more precisely, from the annotated coop. A key strength of the Chicks4FreeID dataset is its expert ground truth labeling for all instances, ensuring reliable verification of results even in open-set Re-ID tasks. This sets it apart from datasets that include completely unannotated instances or rely on ground truth labels generated through clustering or AI methods, which may introduce labeling errors.

**Animal Category** Each instance was assigned to one of 3 animal categories. These are “chicken”, “rooster”, and “duck”. Roosters, and particularly ducks, are exceptions in this dataset, which is primarily composed of female chickens (hens). For simplicity and to align with colloquial language, instances of female chickens were categorized as “chicken” while male chickens were categorized as “rooster”.

**Figure A1:**
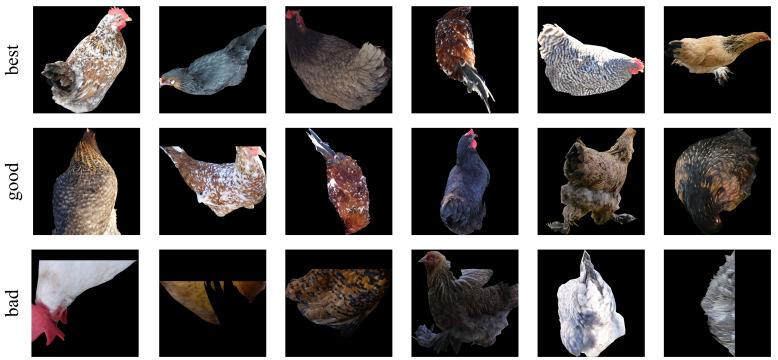
Examples of visibility rating “best”, “good”, and “bad”.

**Visibility Rating** It was common for animals to be partially obscured by other animals or objects in the images. Additionally, they were often not fully contained within the image frame. Acknowledging the resulting varying visibility of the instances, each was assigned one of 3 visibility ratings: “bad”, “good”, or “best” (for examples, see Figure A1; for full overview, see Table A1 and Table A2). The “best” rating includes instances that fully display the animal from the desired top-down perspective and those where only an insignificant part is missing, such as the very tip of the tail feathers. Instances that include only small parts of the animal and with an undesired perspective fall under the “bad” rating. All remaining instances that do not qualify as “bad” or “best” are rated as “good”.

**Coops** The location (one of 11 coops) of each photograph was documented during the capture process. As a result, each image was annotated with the coop it belonged to. It is therefore straightforward to match the animals in the images to their respective coops.

**Semantic Segmentation Masks** Furthermore, 677 color-coded semantic segmentation masks were created, 1 for each image. They include four possible classes: “background”, “chicken”, “duck” and “rooster”. Every mask includes “background” and “chicken”, while “duck” and “rooster” are included if present. While the semantic segmentation masks are not directly involved in the re-ID process, they were added to the dataset due to the availability of existing annotations, expanding the dataset’s utility to support additional semantic segmentation tasks.

### 3.4. Preprocessing

**Figure 2:**
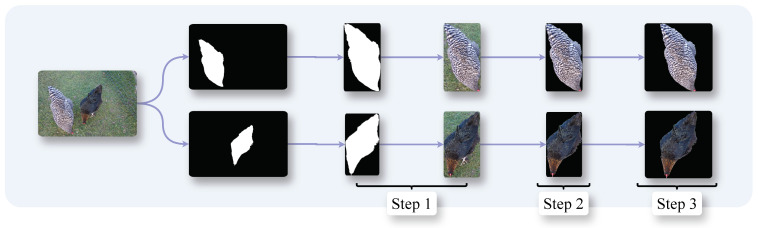
Data preprocessing pipeline for subsequent re-ID.

The Chicks4FreeID dataset contains preprocessed data for the task of re-ID. These are cut-out crops of isolated animal instances with a solid black background. By removing visual cues like feet with rings or environmental features, it is ensured that the re-ID relies solely on the animals’ physical characteristics. The following steps describe the preprocessing procedure to obtain the cut-out crops for the re-ID task. For all individuals captured in an image, a bounding box is created based on the instance masks. In the first step, both the image and the mask are cropped (to the area of interest contained in the bounding box) to focus solely on the individual (see Figure 2: Step 1). The cropped mask is then used to remove the background from the cropped image (Step 2). Finally, the resulting image is adjusted to a square shape for ease of use and consistency (Step 3). The resulting resolutions remain as is, with no resizing taking place.

### 3.5. Dataset Statistics

**Figure 3:**
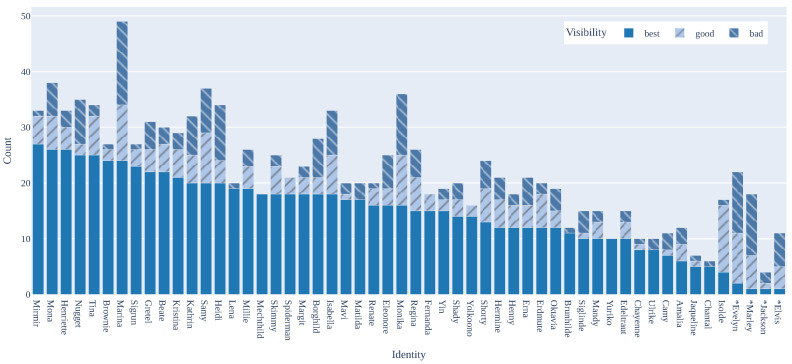
Visibility distributions for all instances of each individual. Ducks and roosters are marked with an asterisk (*).

**Figure A2:**
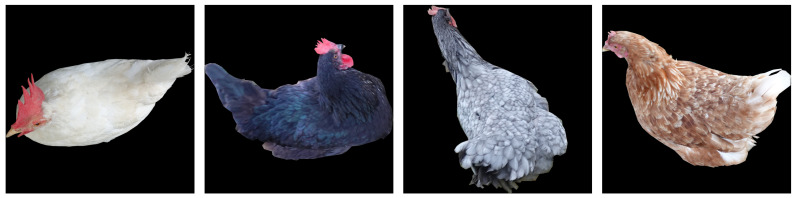
Uniform plumage examples from left to right: solid white, solid black, shades of gray, shades of orange.

The dataset contains 677 images featuring 54 individual animals. The 677 images were captured across 11 different coops, and the 54 individual animals were divided into three categories: “chicken”, “rooster”, and “duck”. There are 50 “chicken”, 2 “rooster”, and 2 “duck” identities. For simplicity and to align with common usage, hens were categorized as “chicken” while the two roosters were given their own category. Each image is paired with a semantic segmentation mask and an instance segmentation mask for each animal depicted. In total, there are 1270 instance segmentation masks. From the instance masks and the corresponding images, 1270 cut-out crops of the individual animals were generated, specifically for use in the re-ID process. There are 1215 “chicken” instances, 15 “rooster” instances, and 40 “duck” instances. The instances also received a visibility rating: 798 “best”, 201 “good”, and 271 “bad” instances. Among the 271 “bad” instances, 69 are labeled “Unknown” as they could not be certainly identified by the annotator. No “Unknown” instances are present among those rated as “best” or “good”. Therefore, the ground truth identity for all instances with these ratings is known. Figure 3 illustrates the number of instances for each individual, along with their visibility ratings. The figure starts with the individual having the most instances rated as “best” and is arranged in descending order. The chicken identity with the most “best” ratings is “Mirmir”, with 27 instances, while “Isolde” has the fewest, with 4 “best” rated instances. The dataset comprises chicken individuals with both uniform and nonuniform plumage. The diversity in appearance possibly aids individual identification, with differences in colors and patterns serving as markers. As a result, this renders the dataset easier to solve than one containing only uniform-plumage individuals. Nevertheless, some individuals in the dataset exhibit uniform plumage, including four that are solid white, four that are solid black, four in shades of gray, and six in shades of orange. Examples of these plumage colors are illustrated in Figure A2. Detailed information about the individuals and the corresponding annotations is provided in Table A3.

## 4. Materials and Methods

### 4.1. Hardware

The training and evaluation were conducted on 64 GB shared memory Apple M3 Max Chips (2023) running PyTorch 2.3.0 with MPS acceleration.

### 4.2. Data

For the experiments, we employed the subset “chicken-re-id-all-visibility”, which features 50 different individuals. It is hosted on Hugging Face [64], a widely used platform for machine learning datasets and models. The dataset encompasses 1146 “chicken” instances of all visibility ratings (793 “best”, 181 “good”, and 172 “bad”). It does not contain any “Unknown” instances nor instances of the animal category “rooster” or “duck”. As a result, it comprises 1146 pairs of preprocessed “chicken” cut-out crops and assigned identities. The subset was split into 916 train pairs and 230 test pairs (stratified). All 50 identities were included in the training set; this ensured that the testing set did not introduce any new identities. For a fair evaluation on all identities, the train/test split was stratified, i.e., each identity had the same fixed percentage of its cut-out crops allocated to the test set. Hence, the test set also included all of the 50 identities. As a consequence of the stratified split, identities with a higher total number of crops contributed more to the test set compared to identities with fewer crops, ensuring proportional representation across all identities. After accessing the described “chicken-re-id-all-visibility”subset, we additionally introduced a validation split (10% of the training set, stratified random split). Consequently, the dataset was finally split into 824 pairs for training (prior to applying any augmentation), 92 pairs for validation, and 230 pairs for testing.

### 4.3. Augmentation

Augmentation was applied dynamically during the training process to the training data: random rotation (360 degrees), random flip (horizontal and vertical), and RandAugment [94]. No data augmentation was applied to the test or validation set. To avoid data leakage, it is important to apply data augmentation only after a train-test split was established. This ensures that augmented versions of the same original image do not appear in both sets.

### 4.4. Feature Extractors

**Vision Transformer** We employed the ViT-B/16 [95] architecture, as implemented in [96]. The hyperparameters (ADAM + CosineWarmup) were inspired by the optimizer used in the lightly benchmarks [97] for their vision transformer backbones.

**Swin Transformer** We also utilized the swin_large_patch4_window12_384 architecture [98] as implemented in [99]. The hyperparameters (Stochastic Gradient Descent + Cosine Annealing, ArcFace [100] loss function) mirrored those used to build the MegaDescriptor, which also employs the same Swin transformer architecture. The Swin transformer itself is based on the vision transformer architecture [95]. The difference between the Swin Transformer and the vision transformer lies in how they handle image data: the Swin transformer uses a hierarchical structure with shifted windows to capture local and global features, while the vision transformer treats images as sequences of patches, relying on self-attention mechanisms throughout.

**MegaDescriptor** The employed MegaDescriptor-L-384 [43] (CC BY-NC 4.0 license [101]) is a state-of-the-art feature extractor for animal re-ID from the WildlifeDatasets toolkit (MIT license). It is based on the Swin transformer architecture [98] and was pretrained on diverse datasets featuring various animal species. A notable hyperparameter choice made by the MegaDescriptor-L384 authors is the ArcFace [100] loss function, which aims to aid in building meaningful embeddings. We selected the frozen MegaDescriptor-L-384 model over DINOv2 [102] and CLIP [103] due to its better performance on unseen animal domains, as reported by the authors. Their evaluation included cattle as an example of an unseen domain [43].

### 4.5. Classifiers

**k-NN** k-NN stores all the training data and calculated distances at the time of prediction. It works by comparing a new chicken’s embedding (feature vector) to all known embeddings. It then finds the k-closest embeddings in the feature space and assigns the most common identity among those neighbors. Our settings were inspired by [104]. We used a high value of k = 200 to provide a broader context, allowing us to consider not just the closest neighbor but the overall composition of the embedding space. For a correct prediction, other individuals must be sufficiently distant from the cluster of the “correct” individual. This k-NN approach prioritizes clustering quality over absolute discriminative power.

**Linear Classifier** To evaluate absolute discriminative power, we relied on a linear classifier. The employed linear classifier is a simple linear layer. It was trained for 90 epochs using the extracted embeddings (feature vectors) of the training data. Thereby, it learned to associate each feature vector with the correct identity by adjusting its weights, ultimately mapping each input to1one of the 50 identity classes. It was validated on the embeddings of the validation set.

### 4.6. Evaluation Metrics

We provide three of the most common metrics for closed set animal re-ID. These are **mAP** (mean average precision), **top-1 accuracy** (ratio of correct predictions versus total predictions), and **top-5 accuracy** (accuracy of the correct class being within the top 5 predictions) as implemented in TorchMetrics [105]. It is important to note that the mAP focuses more on the quality of the probability estimates, serving as a ranking metric that assesses “how well the model assigns high probabilities to the correct chicken.” This is similar to the top-5 accuracy, where the model’s prediction is considered correct if the correct answer is among the top five guesses. However, even though top-5 accuracy can provide some confidence in the model’s predictions, it does not fully capture the model’s actual performance in terms of the number of errors made. Therefore, the top-1 accuracy must be considered the most.

## 5. Experiments

**Figure 4:**
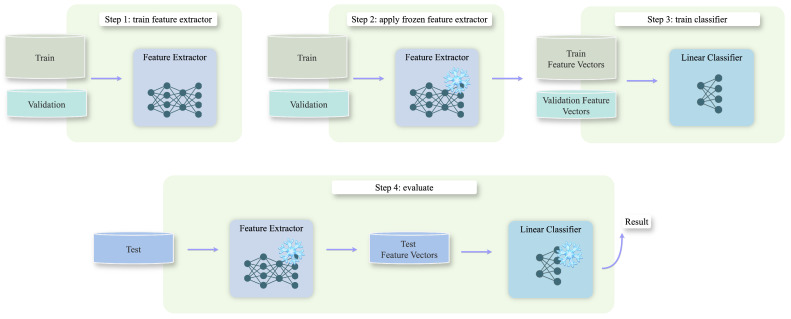
Illustration of the training and evaluation process for the feature extractor and classifier, showcasing the linear classifier as an example in this workflow.

The approach for closed-set re-ID involved two steps. First, a feature extractor generated embeddings for the cut-out crops. Second, the resulting feature vectors (embeddings) were then passed to a classifier to ultimately assign the identities. Feature extractor and classifier were trained separately. Figure 4 illustrates the training and evaluation process. We evaluated four feature extraction models: MegaDescriptor (both frozen and fine-tuned), vision transformer, and Swin transformer. All feature extractors were fed with images at an input resolution of 384 × 384 pixels (the square-shaped cut-out crops were resized). Additionally, we tested with a variation of two classifiers: k-NN and a linear classifier. In our experiments, we explored different training strategies: standard supervised learning (see Section 5.2) and one-shot learning (see Section 5.3). We employed both standard supervised learning and one-shot learning approaches to evaluate performance across different data availability scenarios. The one-shot setting simulated situations with very limited data while also providing insights into generalization capabilities by reducing the risk of overfitting. We also specifically compared the performance of the pretrained MegaDescriptor with its fine-tuned counterpart (see Section 5.1). Each experiment, as a whole, was repeated three times to ensure consistency.

### 5.1. Domain Transfer Experiment

This experiment had two main objectives. First, it aimed to evaluate the performance of the frozen pretrained MegaDescriptor on our Chicks4FreeID dataset, which represents a new, unseen domain. The MegaDescriptor was originally trained on diverse datasets featuring various animal species. However, chickens were not part of the original training data. We evaluated the MegaDescriptor as a feature extractor with its weights frozen, preserving its pretrained state. Second, the experiment sought to enhance performance by fine-tuning the MegaDescriptor on the Chicks4FreeID dataset. This optimally allows the model to adapt by leveraging the features it previously learned from other species and refining them through additional training. For this, we unfroze all the model’s layers and trained it for 200 additional epochs using the training set, as detailed in Section 4.2. For testing and validation, we utilized the test and validation sets, also described in Section 4.2.

### 5.2. Standard Supervised Learning Experiment

This experiment aimed to train a chicken feature extractor from scratch using the Chicks4FreeID dataset. We compared two transformer architectures; the Swin and the vision transformers.

### 5.3. One-Shot Experiment

This experiment aimed to evaluate all previously discussed models in a one-shot learning setting, where the training set was reduced to only one sample per individual. With 50 individuals in the dataset, the training set initially consisted of 50 samples, each representing one individual. All remaining samples of the previously established training set (see Section 4.2) were unused. These 50 samples were then augmented dynamically during training using the methods described in Section 4.3. The validation and test sets remained unchanged, as outlined in Section 4.2. All feature extractors, except the non-fine-tuned frozen MegaDescriptor, were trained on this one-shot training set. The classifiers were also trained/fitted solely on this reduced set. For the non-fine-tuned frozen MegaDescriptor, the only difference in the one-shot setting was therefore the classifier. Among the conducted experiments, this scenario most closely resembled an open-set re-ID setting, where a new, previously undocumented identity appears and initially little data are available. Consequently, the expected performance in an open-set scenario is likely to be similar the results observed in the one-shot setting, particularly for the non-fine-tuned frozen MegaDescriptor. However, the classifier would need to be retrained each time a new individual appears. To enable open-set re-ID and to identify unknown individuals without retraining, the classifier head could be replaced with a clustering algorithm, such as DBSCAN [106,107], or hierarchical clustering [108].

## 6. Results and Discussion

**Figure 5:**
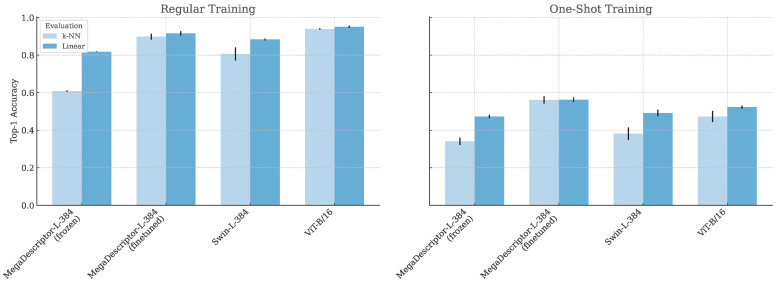
Top-1 accuracy visualized in bar charts. The left bar chart combines the results in Table 2 and Table 3. The right bar chart illustrates the one-shot experiments in Table 4.

**Figure A3:**
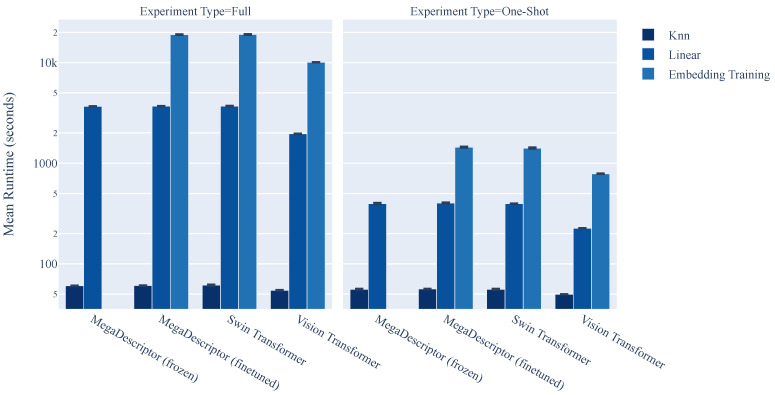
Mean runtime results by experiment type and model (log scale).

Table 2, Table 3 and Table 4 show the results on the test data for the domain transfer, standard supervised learning, and one-shot experiments, respectively. Figure 5 additionally illustrates the top-1 accuracy as bar charts. For runtime results and discussion, see Figure A3 in the Appendix A.

**MegaDescriptor Shows Improved Performance with Fine-Tuning** The fine-tuned MegaDescriptor model achieved a mAP of 96.0% and a top-1 accuracy of 91.6% when paired with a linear classifier (see Table 2). It outperformed the frozen MegaDescriptor in the domain transfer experiment. This aligns with expectations that a model performs not as well on an unseen domain. The fine-tuned MegaDescriptor also outperformed the Swin transformer, which we specifically trained on our data from scratch (see Table 3). This suggests the pretrained features are useful for identifying chickens, since fine-tuning seems preferable to training the same model from scratch.

**Frozen MegaDescriptor is a Powerful Feature Extractor** The frozen MegaDescriptor, when combined with a linear classifier, still performed well, with a mAP of 92.0% and a top-1 accuracy of 81.8% (see Table 2). Its performance was not far behind that of models specifically trained on our data (see Table 3). This is particularly impressive given that the frozen model had no exposure to chicken data. It was trained on a large dataset of many different animal species, which also included bird images. Since birds share common features like feathers, this overlap may have further contributed to the model’s strong performance, in addition to the large training dataset.

**Trained-from-Scratch Vision Transformer Yields the Best Results** The vision transformer trained from scratch, combined with a linear classifier, achieved the overall best results with a mAP of 97.0%, a top-1 accuracy of 95.1%, and a perfect top-5 accuracy of 100.0% (see Table 3).

**Fine-Tuned MegaDescriptor Leads in One-shot Setting** As expected, the performance dropped for all models in the one-shot setting (see Table 4). When trained with only a single image per individual, the fine-tuned MegaDescriptor achieved the best performance, with a 64.5% mAP, a 56.2% top-1 accuracy, and an 81.8% top-5 accuracy, closely followed by the vision transformer, which achieved a 61.9% mAP, a 52.3% top-1 accuracy, and a 80.8% top-5 accuracy. Both models were paired with a linear classifier for these evaluations. The fine-tuned MegaDescriptor likely outperformed the vision transformer in this scenario because it had already learned embeddings through pretraining before being fine-tuned in a one-shot manner. In contrast, the vision transformer had no prior learning, starting from scratch. This pretraining advantage gave the MegaDescriptor a head start in understanding features, likely contributing to its better performance. This underscores that in scenarios with very limited data, like the one-shot example, transfer learning (fine-tuning pretrained models) is more effective than training from scratch. Furthermore, we think that the improved discrimination capabilities make the fine-tuned MegaDescriptor more suitable in an open-set re-ID setting than the other models.

**Evaluation Focus Varies Between Linear Classifiers and k-NN** In all experiments, feature extractors consistently performed worse when paired with k-NN compared to when they were paired with a linear classifier. This was mainly due to the differences in the evaluation focus of the employed classifiers. The employed k-NN approach is more challenging by design as it aims to evaluate the clustering quality in embedding space, instead of absolute discriminative power. Additionally, the k-NN is a parameterless model, while the linear classifier has approximately hidden_dim × 50 parameters. k-NN only captures distances in the feature vector space, whereas a linear classifier can learn robust decision boundaries. Moreover, with a linear classifier, we can track training progress with a validation split, leading to better generalization compared to the fixed boundaries of k-NN.

**Higher-quality Embedding Clusters with Vision Transformer** Training the Swin transformer architecture, which also served as the basis for the MegaDescriptor, appeared to result in lower clustering quality of the embeddings compared to the vision transformer architecture, meaning the overall composition of the embedding space appeared to be better when employing a vision transformer. This difference is evident in Table 2 and Table 3 when paired with k-NN. This could be due to the simpler architecture and loss function, which could lead to reaching better performance faster.

**Higher Accuracy with Vision Transformer** Furthermore, the vision transformer consistently outperformed the Swin transformer when paired with a linear classifier. This could be because it is optimized to predict class probabilities directly, while the Swin transformer focuses on learning the relative distances between features in the feature space. This difference in optimization goals allows the vision transformer to better capture the overall structure of the data, leading to more accurate classification when paired with the same classifier.

## 7. Conclusions

### 7.1. Findings

We introduced the first open-source dataset for chicken re-ID to address the need for well-annotated, publicly available datasets for animal re-ID. Utilizing this dataset, we conducted a series of closed-set re-ID experiments. We combined various transformer-based feature extractors (MegaDescriptor, vision transformer, Swin transformer) with two classifiers (k-NN and linear classifier) and evaluated their performance across different settings (domain transfer, training from scratch, and one-shot learning).

We found that pretraining and domain transfer are critical when dealing with limited data per instance. The MegaDescriptor model proved well suited as a pretrained model in such scenarios. Pretraining on a different domain (e.g., animals other than chickens) enables a model to learn generalizable features that can later be fine-tuned to the specific target domain (e.g., chickens). Interestingly, if sufficient data are available, training a feature extractor from scratch is not necessarily preferable, as the fine-tuned MegaDescriptor outperformed the Swin transformer, which was trained from scratch.

Furthermore, our evaluation suggests that the vision transformer architecture produces higher-quality embedding clusters than the Swin transformer architecture.

Overall, transformer-based architectures as feature extractors achieved high accuracy and mAP on our dataset. The best results on our dataset, however, were achieved with the vision transformer trained from scratch combined with a linear classifier (mAP 97.0%, top-1 95.1%, top-5 100.0%).

### 7.2. Limitations and Future Work

We have not yet explored which features the models prioritize during the identification process, such as patterns, colors, shapes, specific areas of the plumage, or certain body parts. This presents an interesting direction for future research. The classifiers used for the experiments were straightforward and effective for the purposes of this study. While more advanced classifiers could potentially yield even better results, the main focus of this work was on evaluating the feature extractors. Another potential direction for future work is adapting the Chicks4FreeID dataset for open-set Re-ID experiments. Researchers could, for example, exclude certain individuals from the training set and introduce them later during evaluation. The dataset subset used for the experiments contained 1215 instance annotations of 50 individual chickens, offering a compact yet valuable resource. The versioning system of the dataset facilitates potential expansions and continuous improvements, ensuring its ongoing relevance and applicability for future research. For chicken breeds with minimal interindividual variability, such as those with uniform plumage, increasing both the number of individuals and instances per individual could further improve the re-ID process. This expansion would be particularly relevant for applications in industrial farming, where thousands of chickens of a single breed are typically kept. Furthermore, photographing individual chickens over an extended period proved challenging, as free-range chickens are often preyed upon by wild animals such as raccoons, foxes, or rats. Therefore, the decision was made to capture all images of a given individual in a single day. Unfortunately, this approach did not capture changes in appearance over time, such as molting. Future work could focus on increasing variability by photographing chickens under different lighting conditions, such as from dusk till dawn. It could also explore the use of different imaging techniques, like multispectral imaging. Additional improvements could include capturing different angles (front, back, side), documenting various stages of life, and recording changes over time. While challenging, these additions could provide a more comprehensive dataset. However, a dataset gains its true value when it is made publicly available. Therefore, we encourage the release of future datasets under open, unrestricted licenses.

## Figures and Tables

**Table 1:** Publicly available animal re-ID datasets, arranged by date of publication. An asterisk (*) marks data derived from video footage. “n.a.” indicates that a corresponding publication could not be found.

Year	Publ.	Dataset	IDs	Species	Annot.	Avail. at
	ours	Chicks4FreeID	50, 2, 2	chicken, duck, rooster	1215, 40, 15	[64]
2024	[36]	SeaTurtleID2022	438	sea turtle	8729	[48]
2023	[24]	Mammal Club (IISD)	218	11 terrestrial mammal species *	33,612	[65]
2023	[66]	Multi-pose dog dataset	192	dog	1657	[67]
2023	[40]	PolarBearVidID	13	polar bear *	138,363	[68]
2023	[44]	Sea Star Re-ID	39, 56	common starfish, Australian cushion star	1204, 983	[49]
2022	[69]	Animal-Identification-from-Video	58, 26, 9	pigeon *, pig *, Koi fish *	12,671, 6184, 1635	[47]
2022	n.a.	Beluga ID	788	beluga whale	5902	[50]
2022	n.a.	Happywhale	15,587	30 different species of whales and dolphins	51,033	[51]
2022	n.a.	Hyiena ID	256	spotted hyena	3129	[70]
2022	n.a.	Leopard ID	430	African leopard	6805	[71]
2022	[72]	SealID	57	Saimaa ringed seal	2080	[52]
2022	[73]	SeaTurtleIDHeads	400	sea turtle	7774	[53]
2022	n.a.	Turtle Recall	100	sea turtle	2145	[54]
2021	[74]	Cow Dataset	13	cow	3772	[3]
2021	[5]	Cows2021	182	Holstein-Friesian cattle *	13,784	[59]
2021	[75]	Giraffe Dataset	62	giraffe	624	[76]
2021	[13]	iPanda-50	50	giant panda	6874	[77]
2020	[34]	AAU Zebrafish Dataset	6	zebrafish *	6672	[78]
2020	[45]	Animal Face Dataset	1040	41 primate species	102,399	[79]
2020	[32]	ATRW	92	Amur tiger *	3649	[80]
2020	[29]	Lion Face Dataset	94	lion	740	[81]
2020	[82]	NDD20	44, 82	bottlenose and white-beaked dolphin, white-beaked dolphin (underwater) *	2201, 2201	[55]
2020	[29]	Nyala Data	237	nyala	1942	[83]
2020	[6]	OpenCows2020	46	Holstein-Friesian cattle *	4736	[60]
2019	[84]	Bird individualID	30, 10, 10	sociable weaver, great tit, zebra finch	51,934	[46]
2019	[30]	Dog Face Dataset	1393	dog	8363	[85]
2018	[28]	Cat Individual Images	518	cat	13,536	[86]
2018	[87]	Fruit Fly Dataset	60	fruit fly *	2,592,000	[88]
2018	n.a.	HumpbackWhaleID	5004	humpback whale	15,697	[56]
2018	[26]	MacaqueFaces	34	rhesus macaque *	6280	[89]
2017	[4]	AerialCattle2017	23	Holstein-Friesian cattle *	46,340	[61]
2017	[4]	FriesianCattle2017	89	Holstein-Friesian cattle *	940	[62]
2017	[33]	GZGC	2056	plains zebra and Masai giraffe	6925	[90]
2016	[27]	C-Tai	78	chimpanzee	5078	[91]
2016	[27]	C-Zoo	24	chimpanzee	2109	[91]
2016	[2]	FriesianCattle2015	40	Holstein-Friesian cattle *	377	[63]
2015	n.a.	Right Whale Recognition	447	North Atlantic right whale	4544	[57]
2011	[35]	StripeSpotter	45	plains and Grevy’s zebra	820	[35]
2009	[92]	Whale Shark ID	543	whale shark	7693	[58]

**Table 2:** Domain transfer experiment results. The highest scores for each metric are in blue. Rows corresponding to experiments employing a linear classifier are highlighted in gray.

Feature Extractor	Training	Epochs	Classifier	mAP	Top-1	Top-5
MegaDescriptor [43]	pretrained, frozen	-	k-NN	0.563±0.011	0.609±0.006	0.920±0.025
MegaDescriptor [43]	pretrained, frozen	-	linear	0.920±0.008	0.818±0.002	0.976±0.003
MegaDescriptor [43]	pretrained, fine-tuned	200	k-NN	0.835±0.035	0.898±0.026	0.976±0.006
MegaDescriptor [43]	pretrained, fine-tuned	200	linear	0.960 ± 0.009	0.916 ± 0.020	0.982 ± 0.007

**Table 3:** Results of standard supervised learning experiment. Highest scores for each metric are in blue. Rows corresponding to experiments employing a linear classifier are highlighted in gray.

Feature Extractor	Training	Epochs	Classifier	mAP	Top-1	Top-5
Swin Transformer [98]	from scratch	200	k-NN	0.728±0.082	0.806±0.060	0.966±0.013
Swin Transformer [98]	from scratch	200	linear	0.945±0.019	0.884±0.010	0.989±0.004
Vision Transformer [95]	from scratch	200	k-NN	0.923±0.006	0.939±0.006	1.000 ± 0.000
Vision Transformer [95]	from scratch	200	linear	0.970 ± 0.014	0.951 ± 0.009	1.000 ± 0.000

**Table 4:** One-shot learning experiment results. Highest scores for each metric are in blue. Rows corresponding to experiments employing a linear classifier are highlighted in gray.

Feature Extractor	Training	Epochs	Classifier	mAP	Top-1	Top-5
MegaDescriptor [43]	pretrained, frozen	-	k-NN	0.298±0.035	0.341±0.034	0.632±0.037
MegaDescriptor [43]	pretrained, frozen	-	linear	0.522±0.013	0.473±0.015	0.797±0.014
MegaDescriptor [43]	pretrained, fine-tuned	200	k-NN	0.464±0.035	0.561±0.034	0.782±0.022
MegaDescriptor [43]	pretrained, fine-tuned	200	linear	0.645 ± 0.027	0.562 ± 0.022	0.818 ± 0.036
Swin Transformer [98]	from scratch	200	k-NN	0.289±0.057	0.382±0.058	0.620±0.076
Swin Transformer [98]	from scratch	200	linear	0.545±0.039	0.492±0.030	0.726±0.015
Vision Transformer [95]	from scratch	200	k-NN	0.387±0.044	0.473±0.051	0.785±0.013
Vision Transformer [95]	from scratch	200	linear	0.619±0.020	0.523±0.014	0.808±0.012

## Data Availability

The Chicks4FreeID dataset and the accompanying code (excluding imported libraries or models from external sources, which have their own licenses) are released under the CC BY 4.0 license. This license allows for the distribution, remixing, adaptation, and building upon the dataset in any medium or format. Users must give appropriate credit to the authors, include a link to the license, and clearly indicate if any changes were made. Commercial use of the dataset is permitted. Dataset: https://doi.org/10.57967/hf/2345; Code: https://github.com/DariaKern/Chicks4FreeID (accessed on 3 November 2024).

## Abbreviations

The following abbreviations are used in this manuscript:

AI	Artificial Intelligence
k-NN	k-nearest neighbor
mAP	mean average precision
re-ID	reidentification

## Appendix A. Annotations

**Table A1:** Full overview of all “chicken” annotations in the Chicks4FreeID dataset.

Coop	Images	ID	Bad	Best	Good	Total	Coop	Images	ID	Bad	Best	Good	Total
**1**	29	**Coop Total**	**16**	**28**	**5**	**49**	...	...	Camy	3	7	1	11
		#Unknown	11	0	0	11			Samy	8	20	9	37
		Chantal	1	5	0	6			Yin	2	15	2	19
		Chayenne	1	8	1	10			Yuriko	0	10	0	10
		Jaqueline	1	5	1	7	**7**	42	**Coop Total**	**1**	**42**	**5**	**48**
		Mandy	2	10	3	15			Brownie	1	24	2	27
**2**	36	**Coop Total**	**14**	**39**	**13**	**66**			Spiderman	0	18	3	21
		#Unknown	4	0	0	4	**8**	47	**Coop Total**	**2**	**48**	**15**	**65**
		Henny	2	12	4	18			Brunhilde	1	11	0	12
		Shady	3	14	3	20			Fernanda	0	15	3	18
		Shorty	5	13	6	24			Isolde	1	4	12	17
**3**	60	**Coop Total**	**22**	**58**	**16**	**96**			Mechthild	0	18	0	18
		#Unknown	5	0	0	5	**9**	68	**Coop Total**	**14**	**87**	**13**	**114**
		Amalia	3	6	3	12			#Unknown	1	0	0	1
		Edeltraut	2	10	3	15			Mavi	2	17	1	20
		Erdmute	2	12	6	20			Mirmir	1	27	5	33
		Oktavia	4	12	3	19			Nugget	8	25	2	35
		Siglinde	4	10	1	15			Skimmy	2	18	5	25
		Ulrike	2	8	0	10	**10**	140	**Coop Total**	**57**	**189**	**36**	**282**
**4**	26	**Coop Total**	**7**	**29**	**5**	**41**			#Unknown	23	0	0	23
		Hermine	4	12	5	21			Beate	3	22	5	30
		Matilda	3	17	0	20			Borghild	7	18	3	28
**5**	116	**Coop Total**	**84**	**141**	**48**	**273**			Eleonore	6	16	3	25
		#Unknown	22	0	0	22			Henriette	3	26	4	33
		Erna	5	12	4	21			Kristina	3	21	5	29
		Heidi	10	20	4	34			Margit	2	18	3	23
		Isabella	8	18	7	33			Millie	3	19	4	26
		Kathrin	7	20	5	32			Mona	6	26	6	38
		Marina	15	24	10	49			Sigrun	1	23	3	27
		Monika	11	16	9	36	**11**	67	**Coop Total**	**8**	**80**	**13**	**101**
		Regina	5	15	6	26			Gretel	5	22	4	31
		Renate	1	16	3	20			Lena	1	19	0	20
**6**	46	**Coop Total**	**16**	**52**	**12**	**80**			Tina	2	25	7	34
		#Unknown	3	0	0	3			Yolkoono	0	14	2	16
...	...	...	...	...	...	...	**Total**	**677**	**50**	**241**	**793**	**181**	**1215**

**Table A2:** Full overview of all “rooster” and “duck” annotations in the Chicks4FreeID dataset.

Coop	ID	Category	Bad	Best	Good	Total
**4**	**Coop Total**		**22**	**3**	**15**	**40**
	Evelyn	Duck	11	2	9	22
	Marley	Duck	11	1	6	18
**5**	Elvis	Rooster	6	1	4	11
**9**	Jackson	Rooster	2	1	1	4
**Grand Total**	**4**		**30**	**5**	**20**	**55**

## Appendix B. Plumage

**Table A3:** Overview of uniform plumage “chicken” individuals in the Chicks4FreeID dataset. Grand total of annotations of mixed plumage individuals is included for completeness.

Plumage	ID	Coop	Bad	Best	Good	Total
**Solid White**	**Total**		**5**	**28**	**5**	**38**
	Chantal	1	1	5	0	6
	Chayenne	1	1	8	1	10
	Jaqueline	1	1	5	1	7
	Mandy	1	2	10	3	15
**Solid Black**	**Total**		**5**	**39**	**21**	**65**
	Erdmute	3	2	12	6	20
	Ulrike	3	2	8	0	10
	Isolde	8	1	4	12	17
	Fernanda	8	0	15	3	18
**Shades of Gray**	**Total**		**8**	**66**	**10**	**84**
	Erna	5	5	12	4	21
	Mavi	9	2	17	1	20
	Sigrun	10	1	23	3	27
	Yolkoono	11	0	14	2	16
**Shades of Orange**	**Total**		**16**	**90**	**17**	**123**
	Henny	2	2	12	4	18
	Shady	2	3	14	3	20
	Shorty	2	5	13	6	24
	Brunhilde	8	1	11	0	12
	Mechthild	8	0	18	0	18
	Gretel	11	5	22	4	31
**Uniform Plumage**	**Grand Total**		**34**	**223**	**53**	**310**
**Mixed Plumage**	**Grand Total**		**207**	**570**	**128**	**905**

## Appendix C. Runtime

For efficiency and performance comparison, we report runtime speeds for each run in Figure A3.

Embedding training runtime and classifier training runtime are reported separately. Since each experiment was repeated three times, we report the mean values and error bars for each distinct experiment group. k-NN was the fastest classifier, taking around 1 min per run. This was consistent across both one-shot and full experiments, indicating that runtime speed was primarily dominated by overhead. Overall, excluding k-NN, training one-shot models was significantly faster than training with full datasets, by one order of magnitude, as seen in the logarithmic scale. The figure also shows that training only the classifiers (k-NN and linear) was much more efficient than training or fine-tuning the full pipeline. Training the comparatively large feature extractors took considerably longer. Another noticeable gap exists between the vision transformer and MegaDescriptor architectures (see Embedding Training bars for MegaDescriptor (fine-tuned) and Swin transformer versus vision transformer). This difference arises because the vision transformer avoids calculating shifting windows and uses a simpler loss function that requires only one image instead of two.
